# The use of Lutetium-177 PSMA radioligand therapy with high dose rate brachytherapy for locally recurrent prostate cancer after previous definitive radiation therapy: a randomized, single-institution, phase I/II study (ROADSTER).

**DOI:** 10.1186/s12885-023-10851-0

**Published:** 2023-04-20

**Authors:** Lucas C. Mendez, Aneesh Dhar, David Laidley, Madeleine Moussa, Jose A. Gomez, Joseph Chin, T-Y Lee, Jonathan D. Thiessen, Douglas Hoover, Kathleen Surrey, Joelle Helou, Vikram Velker, Rohann J. Correa, David D’Souza, Jane Bayani, Glenn Bauman

**Affiliations:** 1 London Health Sciences Centre, London, Ontario Canada; 2 Lawson Health Research Institute, London, Ontario Canada; 3 Department of Oncology, Western University, London, Ontario Canada; 4 Department of Medical Biophysics, Western University, London, Ontario Canada; 5 Ontario Institute for Cancer Research, Toronto, Ontario Canada

**Keywords:** Prostate Cancer, Isolated Local Failure, Radiorecurrent Prostate Cancer, High Dose Rate Brachytherapy, Lutetium-177, Prostate Specific Membrane Antigen, Positron Emission Tomography, Radioligand Therapy

## Abstract

**Background:**

Isolated local failure (ILF) can occur in patients who initially receive definitive radiation therapy for prostate cancer. Salvage therapy for ILF includes high dose rate (HDR) brachytherapy. Prostate Specific Membrane Antigen (PSMA) Positron Emission Tomography (PET) can accurately detect ILF and can exclude extraprostatic disease. Lutetium-177 PSMA Radioligand Therapy (RLT) is a novel treatment for prostate cancer that can target prostate cancer accurately, while sparing radiation dose to normal tissues.

**Methods:**

ROADSTER is a phase I/II randomized, single-institution study. Patients with an ILF of prostate cancer after definitive initial radiation therapy are eligible. The ILF will be confirmed with biopsy, magnetic resonance imaging (MRI) and PSMA PET. Patients will be randomized between HDR brachytherapy in two fractions (a standard of care salvage treatment at our institution) (cohort 1) or one treatment of intravenous Lutetium-177 PSMA RLT, followed by one fraction of HDR brachytherapy (cohort 2). The primary endpoints for the phase I portion of the study (n = 12) will be feasibility, defined as 10 or more patients completing the study protocol within 24 months of study activation; and safety, defined as zero or one patients in cohort 2 experiencing grade 3 or higher toxicity in the first 6 months post-treatment. If feasibility and safety are achieved, the study will expand to a phase II study (n = 30 total) where preliminary efficacy data will be evaluated. Secondary endpoints include changes in prostate specific antigen levels, acute toxicity, changes in quality of life, and changes in translational biomarkers. Translational endpoints will include interrogation of blood, urine, and tissue for markers of DNA damage and immune activation with each treatment.

**Discussion:**

ROADSTER explores a novel salvage therapy for ILF after primary radiotherapy with combined Lutetium-177 PSMA RLT and HDR brachytherapy. The randomized phase I/II design will provide a contemporaneous patient population treated with HDR alone to facilitate assessment of feasibility, tolerability, and biologic effects of this novel therapy.

**Trial registration:**

NCT05230251 (ClinicalTrials.gov).

## Background

Prostate cancer is a major male health issue, with over 1.4 million new cases of prostate cancer diagnosed and 375,000 new prostate cancer deaths in 2020 worldwide [1]. Many patients may choose radiation therapy for treatment of prostate cancer but despite improvements in treatment, progression or recurrence can occur, with approximately 20–47% of men having a rise of prostate specific antigen (PSA) that meets the Phoenix criteria for biochemical failure (BCF) at 15 years post-treatment [2, 3]. One scenario of clinical importance is isolated local failure (ILF), without evidence of metastatic disease. In a meta-analysis of patients with prostate cancer recurrence post-radiation, at a median follow-up of 11 years, there was a 7.2% and 13% ILF rate for intermediate-risk and high-risk prostate cancer, respectively [4]. In this analysis, while most patients developed metastases from clinically recurrence-free state, the presence of ILF was associated with worse metastasis-free survival (MFS) in intermediate- and high-risk patients, and worse prostate cancer-specific survival and overall survival (OS) in high-risk patients. Furthermore, there was some evidence that a “second wave” of distant metastases could result after ILF in these patients.  Local failure in these trials was defined using clinical examination and conventional imaging and likely underestimates the proportion of men with ILF.

There are several potentially curative treatment options for patients with ILF, including salvage radical prostatectomy (RP), as well as prostate directed ablative therapies such as cryotherapy, high intensity focused ultrasound (HIFU), and salvage radiotherapy options such as stereotactic body radiation therapy (SBRT), low dose rate (LDR) brachytherapy or high dose rate (HDR) brachytherapy. In a recent meta-analysis comparing these options, the chances of recurrence-free survival (RFS) were similar, with approximately half of patients with durable disease control with the different salvage techniques [5]. In a multi-institutional, prospective trial assessing the role of LDR brachytherapy in a highly selected population, the 10-year BCF rate was 46%, with only 5% of the patients having signs of ILF after salvage brachytherapy [6]. Collectively, these results suggest that a significant proportion of patients with presumed ILF on conventional staging scans (computed tomography, bone scintigraphy, magnetic resonance imaging) at the time of salvage therapy may already have disease outside the prostate.

Prostate-specific membrane antigen positron emission tomography (PSMA PET) has been used more recently as a tool to detect prostate cancer recurrence after radiation treatments. In this setting, PSMA PET has been shown to change the management plan in a majority of patients when compared to the management plan based on conventional imaging alone [7]. By identifying men with extra-prostatic disease not evident on conventional imaging, PSMA PET/CT could result in better selection of patients with true ILF. In addition to using PSMA PET for the diagnosis of prostate cancer, PSMA-targeting radioligand therapies (RLTs) have been developed and have shown benefit in the metastatic, castrate-resistant prostate cancer (CRPC) setting. In particular, Lutetium-177 (Lu-177) PSMA RLT has been shown to improve survival outcomes in the PSMA PET positive, CRPC setting and results in significant cytolytic activity and PSA response [8].

Lu-177 PSMA RLT emits beta particles with a maximum energy of 497 keV, which limits the range of these particles in tissue to less than 2 mm [9]. Most studies report a delivered dose to the tumor of 2–6 Gy per GBq of Lu-177 PSMA RLT administered, although this dose can range between 1.4 and 14.5 Gy per GBq [10]. In comparison, the dose delivered to the kidneys, bone marrow, salivary glands, and lacrimal glands has been reported as 0.4-1.0 Gy per GBq, 0.01–0.1 Gy per GBq, 0.6–1.4 Gy per GBq, and 1.0-2.8 Gy per GBq, respectively. Thus, a single dose of 6.8 GBq could provide doses to ILF in a range similar to that delivered with a fraction of HDR brachytherapy with anticipated low dose to organs at risk that are typically limiting in the setting of salvage radiotherapy, i.e., the bladder and rectum.

We hypothesized that a theragnostic combination targeting PSMA may improve outcomes and reduce toxicity in men with suspected ILF after definitive radiotherapy. Specifically, use of a diagnostic PSMA-targeting agent will improve our ability to select men with a high probability of ILF. The use of a PSMA-targeting RLT would then allow the selective treatment of the site of ILF as well as potential extra-prostatic disease not detectable even with the diagnostic PSMA PET imaging. In ROADSTER, we aim to assess safety and feasibility of adding Lu-177 PSMA RLT in combination with HDR brachytherapy for ILF after definitive radiotherapy. A randomized phase I/II design will be used to provide a contemporaneous cohort of men treated with our institutional standard salvage therapy of two fractions of HDR. Translational endpoints will include acquisition of liquid and tissue biospecimens for the assessment of response biomarkers and assessment of DNA damage and immune activation among the two cohorts.

## Methods

### Inclusion and exclusion criteria

Patients with suspected ILF after radiation will be recruited. Patients must have a BCF per Phoenix criteria at least two years after initial prostate cancer treatment, an intra-prostatic lesion with PSMA PET avidity (Standard Uptake Value (SUV) of 3.0 or greater on a diagnostic PSMA PET/CT), and no evidence of extra prostatic disease.

All patients are required to have a prostate biopsy to confirm ILF at the time of enrolment and have no contraindications for HDR brachytherapy, general anaesthesia, PSMA PET/MRI or Lu-177 PSMA RLT. Contraindications for these procedures include having inadequate marrow function (absolute neutrophil count less than 1.5 × 10^9^/L, platelet count less than 100 × 10^9^/L, hemoglobin less than 90 g/L, or transfusions required in the previous two weeks prior to enrolment); inadequate renal function (estimated creatinine clearance less than 30 mL/min by Cockroft Gault equation); or inadequate hepatic function (total bilirubin greater than 1.5 times the upper limit of normal (ULN) or the alanine aminotransferase greater than 3.5 times the ULN). Patients must not have any ongoing grade 3 or greater GU or GI toxicity (by Common Terminology Criteria for Adverse Events (CTCAE) v4.0) associated with their initial prostate cancer treatment. Patients with previous ablative prostate radiotherapy, i.e., HDR brachytherapy, LDR brachytherapy or SBRT to the prostate, will be excluded. Concurrent or adjuvant ADT at the time of salvage therapy is not permitted; patients given ADT as part of the original primary radiotherapy are eligible.

### Baseline assessment

Patients will be required to complete baseline assessments of toxicity by CTCAE v4.0, quality of life (QoL) related to prostate cancer by the Expanded Prostate Cancer Index Composite (EPIC), and baseline bloodwork to assess marrow, renal and hepatic function. Baseline bloodwork and urine samples will be collected as liquid biomarkers for future biomarker analysis. A hybrid PSMA PET/MRI will be done prior to randomization. As part of the PET/MRI, delayed PET images of the pelvis will be acquired at least 60 min after the PSMA ligand injection. MR images of the pelvis will be acquired, including T2 weighted, diffusion weighted, and dynamic contrast enhanced sequences. Imaging will be read by a certified Nuclear Medicine specialist (D.L.) experienced in PSMA PET/MRI and PET/CT interpretation using established interpretation schemes [11, 12]. The MR images will be used for intra-operative HDR guidance for both tissue sampling and selective HDR brachytherapy boosting of the foci of ILF.

### Treatment

**Fig. 1:**
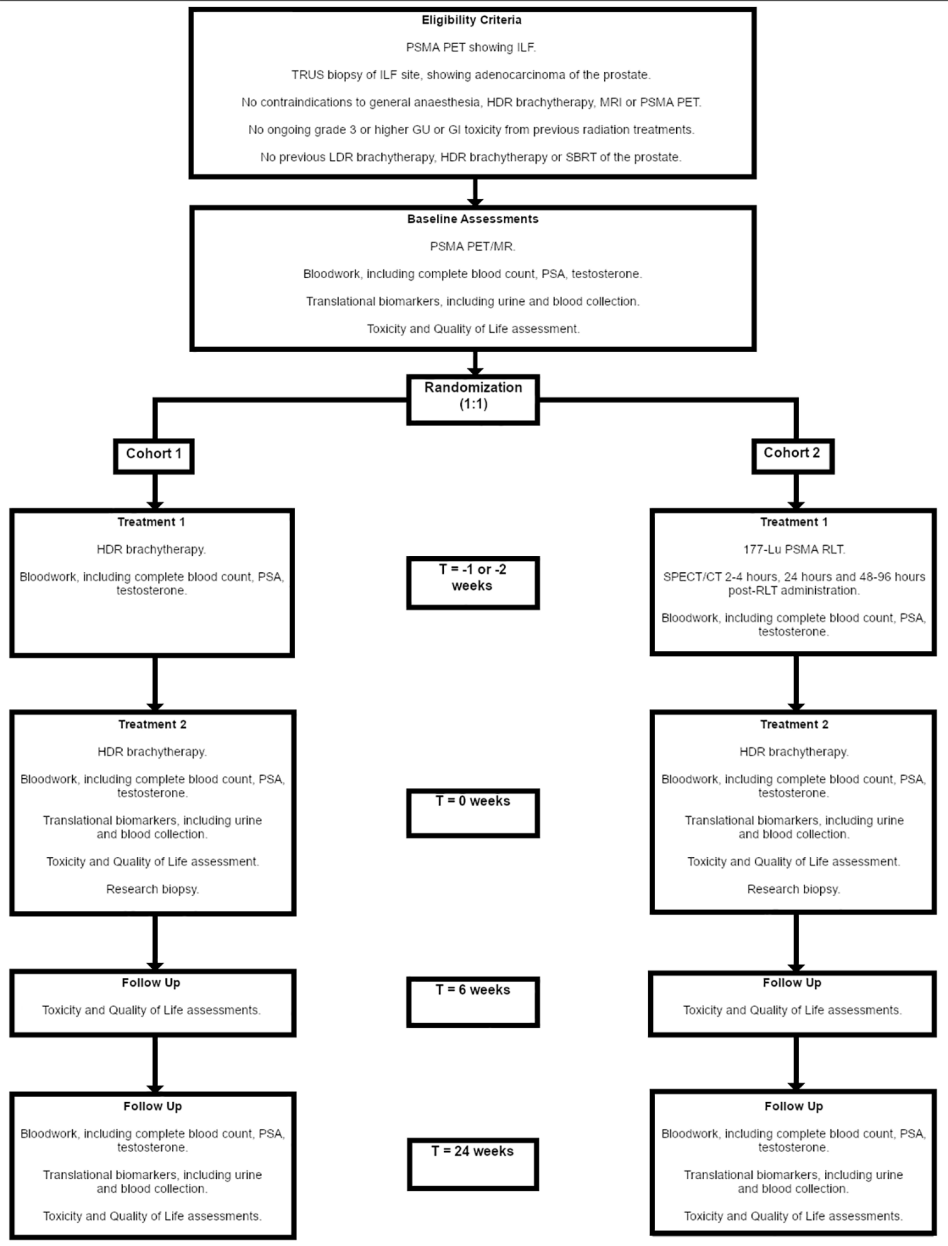
ROADSTER trial schema

The trial schema is given in Fig. 1. Patients will be randomized between cohort 1, consisting of two separate whole-gland plus focal boost prostate HDR brachytherapy treatments (considered a standard salvage treatment in our institution) or cohort 2, consisting of one treatment of Lu-177 PSMA RLT, followed by one treatment of prostate HDR brachytherapy. In both cohorts, the time between the first treatment and the second will be 1–2 weeks.

Brachytherapy will be performed using transrectal ultrasound (TRUS) guidance, under general anesthesia in a dedicated brachytherapy suite, with intraoperative planning. Pre-procedure PSMA PET/MR images will be fused with intra-operative TRUS images using an in-house developed surface-based deformable algorithm to accurately delineate the areas of ILF. For each HDR brachytherapy treatment, 10.5 Gy will be prescribed to the entire prostate, with a focal boost to 13.5 Gy to the clinical target volume of the primary tumor (CTVp), as defined by the gross tumor volume (GTV) on all available images with a 5 mm expansion to account for microscopic disease. Details on brachytherapy planning objectives are found in Table 1.

**Table 1:** Dose objectives for a single HDR brachytherapy treatment

Structure	Dose parameter^1^	Objective (Acceptable)
Prostate	V 90%	Greater than 99% (95%).
	V 100%	Greater than 95% (90%).
	V 150%	Less than 35% (40%).
	V 200%	Less than 11% (15%).
CTVp^2^	V 90%	Greater than 99% (95%).
	V 100%	Greater than 95% (90%).
Urethra	D 0.01 mL	Less than 12 Gy.
Rectum	D 0.1 mL	Less than 8.5 Gy (9 Gy).
	D 1 mL	Less than 6.5 Gy (7 Gy).

^*1*^ *The dose parameter “V X%” refers to the proportion of the structure’s volume receiving at least X% of the prescription dose. The dose parameter “D Y mL” refers to the dose received by the hottest Y mL of the structure.*

^*2*^ *The clinical target volume of the primary tumor (CTVp) is constructed by taking the union of the GTVs, as delineated on MR and PSMA PET, expanding the structure by 5 mm uniformly, then editing the resulting structure to the boundary of the prostate and the urethra.*

Lu-177 PSMA RLT will be delivered in the Department of Nuclear Medicine as per local practice. One dose of 6.8 GBq of Lu-177 PSMA RLT will be administered intravenously, which will deliver an estimated tumor dose of 13.6–40.8 Gy, given the estimated dose of 2–6 Gy per GBq that has been reported in the literature [10]. Patients will be monitored in an outpatient setting afterwards to assess for adverse events and will undergo Single Photon Emission Computerized Tomography (SPECT) / CT scans at three time points: 2–4 h, 24 h, and 48–96 h post Lu-177 PSMA RLT administration for dynamic dose quantification.

**Fig. 2:**
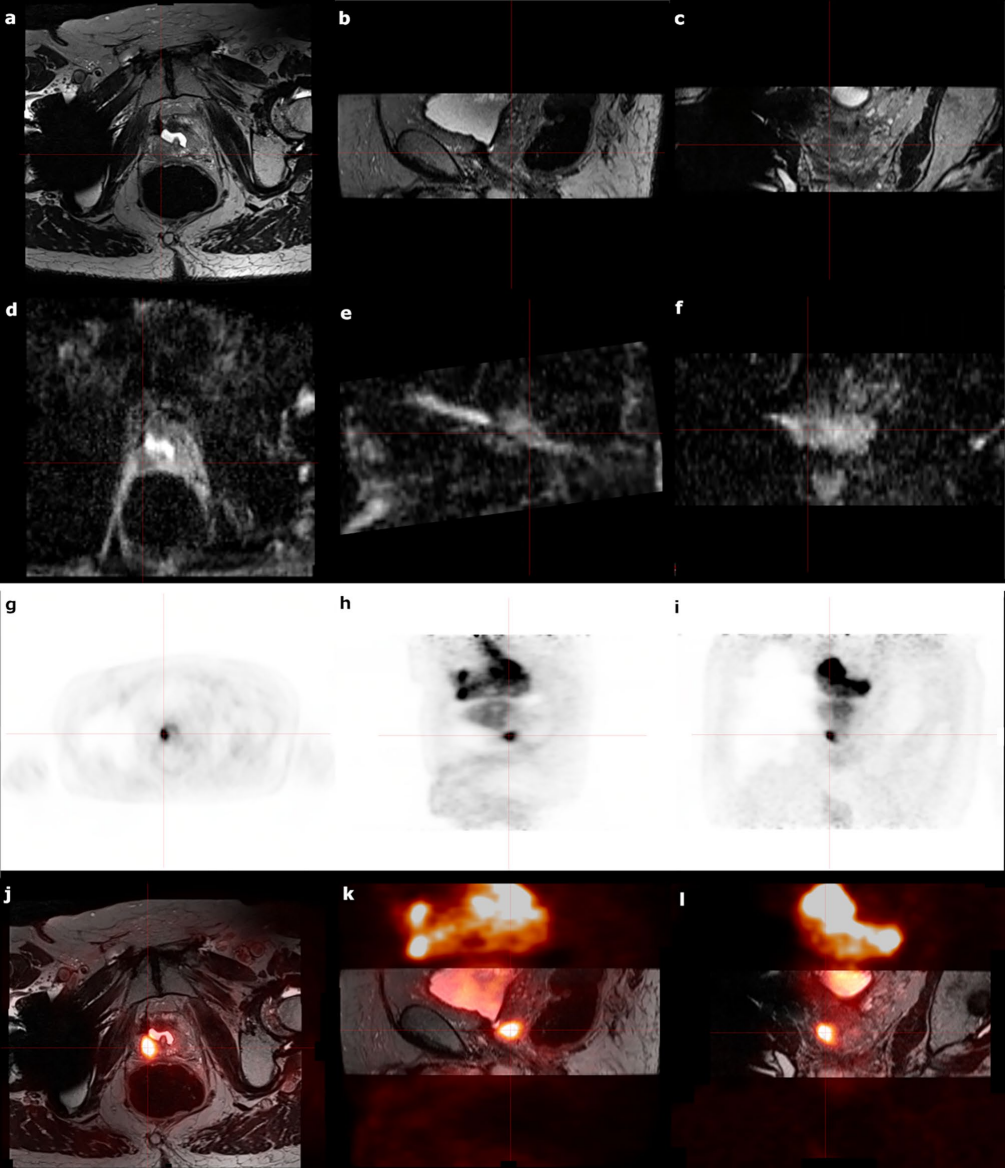
Representative images of the first ROADSTER patient enrolled on cohort 2 Figures **2a** and **2b**, and **2c** show the axial, sagittal and coronal views of the T2-weighted MR images. Figures **2d**, **2e**, and **2f** show axial, sagittal and coronal views for the ADC maps. Figures **2g**, **2h** and **2i** represent the axial, sagittal and coronal views of the avidity of the LF on PSMA PET. Figures **2j**, **2k** and **2l** show the axial, sagittal and coronal views of the fusion of the PSMA PET images to the T2-weighted MR images. Of note, the patient did have a previous transurethral resection of the prostate that was not deemed to be a contraindication to salvage HDR brachytherapy.

**Fig. 3:**
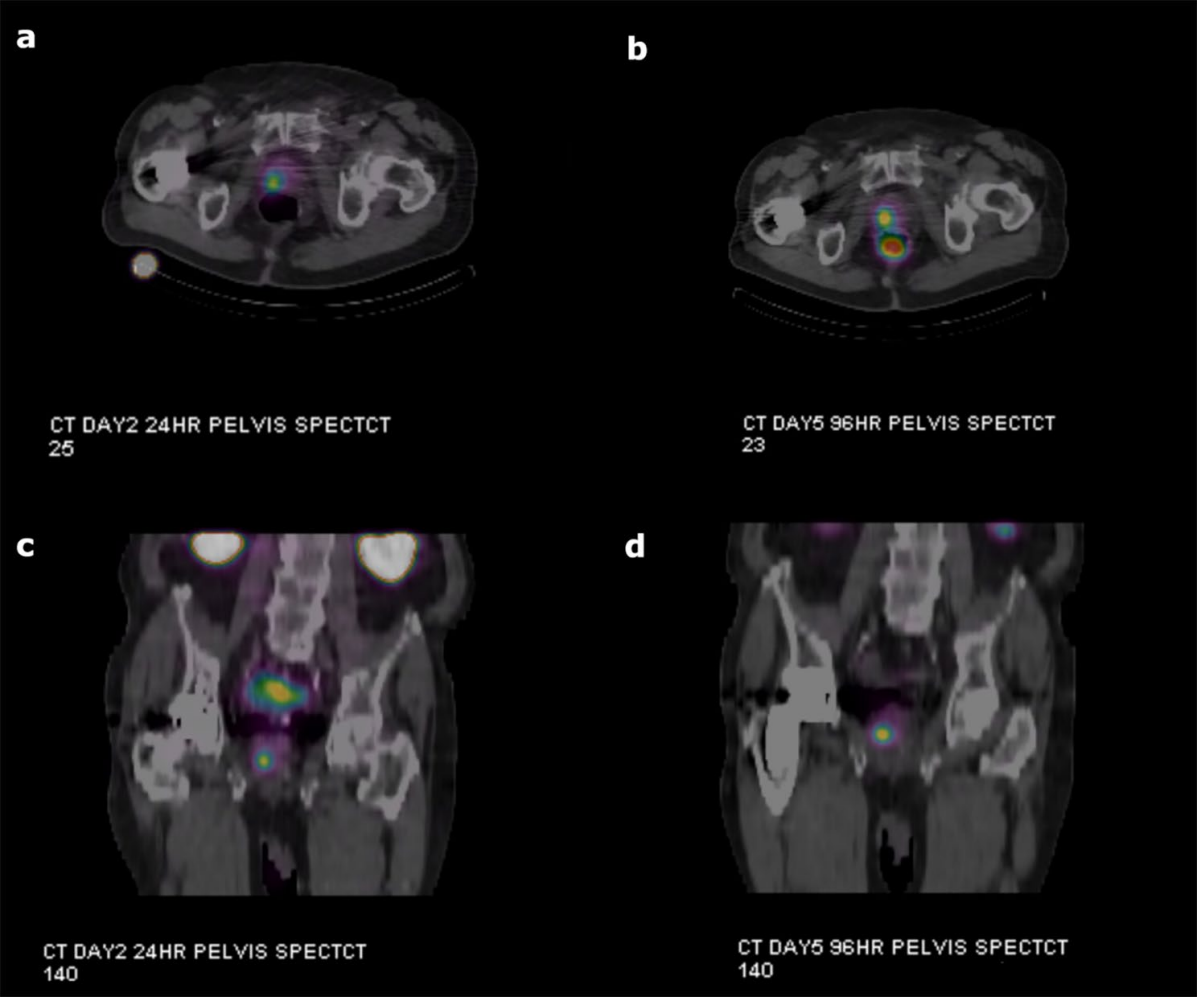
Post Lu-177 PSMA RLT SPECT/CT images Figures **3a** and **3b** show the axial views of the SPECT/CT images at 24 and 96 h post Lu-177 PSMA RLT administration. Figures **3c** and **3d** show the coronal views of the SPECT/CT images at 24 and 96 h post Lu-177 PSMA RLT administration. There was no uptake in the prostate on the SPECT/CT images taken 4 h after the Lu-177 PSMA RLT administration

Figure 2 shows images from the first patient enrolled in cohort 2 of the ROADSTER trial, including the T2-weighted MR images and apparent diffusion coefficient (ADC) maps, PSMA PET images and PSMA PET images fused to the T2-weighted MR images. Figure 3 shows the post Lu-177 PSMA RLT SPECT images for this patient.

### Primary and secondary endpoints

ROADSTER is a phase I/II, randomized controlled trial with safety and feasibility as primary outcomes. The planned accrual for the phase I portion of the study will be 12 patients: six patients in cohort 1 and six patients in cohort 2. Safety of each cohort will be declared if no more than one patient in each cohort experiences a grade 3 or higher CTCAE V4.0 toxicity at 6 weeks or 6 months post-treatment. Feasibility will be declared if 10 or more of the planned 12 patients are successfully accrued and complete the study as per protocol within 24 months of activation. If the initial accrual of 12 men across both cohorts demonstrates both safety and feasibility, the study will expand to the phase II portion, in which 30 men in total will be enrolled and randomized between these two cohorts. Sample sizes were chosen to support measuring the safety and feasibility of this study in the phase 1 and 2 portions. No formal statistical sample size calculation was performed.

Secondary endpoints will include biochemical response, defined as a decrease of PSA by 50% from the baseline level; acute toxicity, defined by changes in CTCAE v4.0 scores; and changes in EPIC QoL scores. These secondary endpoints will be assessed at baseline, 6 weeks after treatment and 6 months after treatment.

### Translational exploratory endpoints

At the time of the second HDR treatment for cohort 1 and the HDR treatment for cohort 2, patients will receive a research prostate biopsy via a transperineal approach under ultrasound guidance. Targeted biopsies of PSMA PET/MR defined lesions will be obtained along with systematic biopsies of the whole gland. These tissue samples are obtained for further translational studies and will assess the effects of HDR brachytherapy in cohort 1 and Lu-177 PSMA RLT in cohort 2 on the prostate cancer ILF.

Blood and urine samples will be collected prior to the first treatment, prior to the second treatment, at 6 weeks after the second treatment and at 6 months after the second treatment, in both cohorts 1 and 2. Patients will have serum PSA, marrow function, renal function and hepatic function bloodwork at 6 months after the second treatment in both cohorts. CTCAE v4.0 toxicity scores and EPIC QoL scores will be done at 6 weeks and 6 months after the second treatment as well.

Regarding the translational biomarkers, the research biopsy specimens will undergo panel-based deoxyribonucleic acid (DNA) sequencing analysis of commonly altered DNA repair pathway and prostate cancer genes, tumor-associated T-cell receptor deep-sequencing (TCR-seq), as well as PSMA expression and gamma-H2A histone family member X (gamma-H2AX) expression, a marker of radiation-induced DNA damage, by immunohistochemistry. Blood plasma will also undergo circulating tumor DNA (ctDNA) sequencing as well as peripheral blood mononuclear cell (PBMC) analysis with TCR-seq and flow cytometry assessing markers of immune activation and radiation-induced DNA damage (e.g., gamma-H2AX). Finally, urine will be collected for urine tumor DNA (utDNA) sequencing.

## Discussion

As a primary outcome, the ROASDTER trial investigates the feasibility and safety of a novel salvage strategy that combines Lu-177 PSMA RLT with HDR brachytherapy in patients with proven radiorecurrent ILF with no signs of metastatic disease. By adding Lu-177 PSMA RLT as a systemic therapy early in the course of cancer relapse, occult metastatic disease may be treated early, potentially reducing rates of systemic progression, which is the most frequent pattern of relapse in this setting [6]. Further, while there could be some systemic toxicity related to the administration of Lu-177 PSMA RLT, including fatigue, xerostomia, dry eyes, decreased blood counts, reduced renal function, nausea, vomiting, diarrhea, constipation, arthralgias, and anorexia, our expectation is that this treatment combination will be well tolerated, and the risk of adverse effects will be minimized by the single dose administration. [8, 10]. Additionally, Lu-177 PSMA RLT allows for greater dose fall-off when compared to brachytherapy (beta-emitter with less than 2 mm tissue penetration) and possibly allows for improved OARs sparing and more selective radiation dose escalation based on the uptake of the radioligand.

Given the physical and biological characteristics of Lu-177 PSMA RLT, we hypothesize this may have an improved toxicity profile compared with other salvage therapy options. Specifically, in a comprehensive meta-analysis of salvage therapy options, there were lower severe genitourinary (GU) toxicity rates amongst the radiation salvage options compared with the non-radiation options. The severe GU toxicity rates for SBRT, LDR brachytherapy, and HDR brachytherapy were 5.6%, 9.1%, and 9.6%, respectively, while the severe GU toxicity rates for cryotherapy, RP and HIFU were 15%, 21%, and 23%, respectively [5]. Severe gastrointestinal (GI) toxicity rates were low in all treatment options, with SBRT and HDR brachytherapy having 0.0% severe GI toxicity each, and HIFU, cryotherapy, RP and LDR brachytherapy having a rate of 0.8%, 0.9%, 1.5% and 2.1%, respectively. Similarly, in a prospective trial of LDR brachytherapy used for salvage in radiorecurrent prostate cancer, there was a late grade 3 combined GU and GI toxicity rate of 14%, with no grade 4 or 5 adverse events [13].

As a secondary outcome, this trial investigates disease control associated with a standard salvage treatment option, involving two fractions of prostate HDR brachytherapy, in the PSMA-PET era. Most of the salvage data in the literature precedes PSMA PET use and the ROADSTER trial may provide a new benchmark by using PSMA PET as a screening tool to select patients with lower likelihood of having any occult extra-prostatic disease at the time of diagnosing the ILF. This “stage migration” effect may allow for patients who are included in the trial to have a more favourable recurrence-free survival, with adequate treatment of their ILF site. Moreover, PSMA PET and MR data together may allow for more accurate definition of the recurrent disease in the prostate at the time of HDR prostate brachytherapy boost with a focused boost to the lesion and better OAR sparing [14, 15]. The ROADSTER trial will provide a cohort of men treated with an institutional standard salvage therapy with two fractions of HDR brachytherapy. A contemporaneous comparison of the two cohorts in this trial can help account for the potential bias introduced by the incorporation of diagnostic PSMA PET into patient selection and treatment delivery.

Additional outcomes in ROADSTER trial include translational studies, assessing the effects of Lu-177 PSMA RLT and HDR brachytherapy on the radiorecurrent prostate cancer. Tissue, blood, and urine biospecimens will be collected at during the study. These biospecimens will be interrogated to characterize effects on tumor and normal tissue radiation injury and immune activation and to assess for potential biomarkers of response or toxicity after Lu-177 PSMA RLT and HDR brachytherapy.

While out of scope for the current trial protocol, which is focused on safety and feasibility endpoints, our practice for men receiving salvage brachytherapy includes long term biochemical and clinical monitoring. Future plans will include examination of long-term disease control and side effects in this population.

While there are several Lu-177 PSMA RLT trials ongoing for patients with CRPC, there are only a few studies that are incorporating Lu-177 PSMA RLT in metastatic castrate sensitive prostate cancer (CSPC): (NCT04720157; NCT04443062; NCT05079698; NCT04343885; and NCT03828838) [16]. Further to these, in the LUNAR study (NCT05496959), patients with oligorecurrent prostate cancer (1–5 extraprostatic lesions on diagnostic PSMA PET) will be randomized between SBRT to all lesions compared to SBRT to all lesions plus two treatments of Lu-177 PSMA RLT, administered 112 days and 56 days prior to the start of SBRT. In another trial, PROQURE-1 (NCT05162573), patients with high-risk, node positive CSPC receive EBRT to the prostate and pelvis and 36 months of concurrent and adjuvant ADT, along with either 3, 6 or 9 GBq of Lu-177 PSMA RLT as a one-time treatment during their second week of EBRT. In this phase I trial, patients will be assessed for their maximum tolerated dose of Lu-177 PSMA RLT. Finally, there are also two trials that involve Lu-177 PSMA RLT administered prior to radical prostatectomy (NCT04430192; NCT04297410). To our knowledge, ROADSTER is the first trial to propose the safety and utility of Lu-177 PSMA RLT in patients with ILF after prostate radiation.

In summary, the ROADSTER trial is a randomized phase I/II trial investigating safety, feasibility and early efficacy of a novel salvage treatment strategy involving Lu-177 PSMA RLT in conjunction with whole prostate HDR brachytherapy with a focused boost. The randomized phase I/II design will provide a contemporaneous patient population treated with HDR alone to facilitate assessment of feasibility, tolerability, and biologic effects of this novel therapy. Access to MR and PSMA PET imaging will allow for localization of the recurrent disease within the prostate, guidance of focal boost delivery at the time of HDR brachytherapy and the precision acquisition of tissue biospecimens from involved and uninvolved prostate for translational science correlative studies.

## Data Availability

The datasets used and/or analysed during the current study are available from the corresponding author on reasonable request.

## List of Abbreviations

ADC: Apparent Diffusion Coefficient
ADT: Androgen Deprivation Therapy
BCF: Biochemical Failure
CRPC: Castrate Resistant Prostate Cancer
CSPC: Castrate Sensitive Prostate Cancer
CT: Computed Tomography
CTCAE v4.0: Common Terminology and Criteria for Adverse Events version 4.0
ctDNA: Circulating Tumor Deoxyribonucleic Acid
CTVp: Clinical Target Volume of the Primary Tumor
DNA: Deoxyribonucleic Acid
DRE: Digital Rectal Exam
EBRT: External Beam Radiation Therapy
EPIC: Expanded Prostate Cancer Index Composite
Gamma-H2AX: Gamma H2A Histone Family Member X
GBq: Gigabecquerel
GI: Gastrointestinal
GTV: Gross Tumor Volume
GU: Genitourinary
Gy: Gray
HDR: High Dose Rate
HIFU: High Intensity Focused Ultrasound
ILF: Isolated Local Failure
LDR: Low Dose Rate
Lu-177: Lutetium-177
MFS: Metastasis-Free Survival
MRI: Magnetic Resonance Imaging
OARs: Organs at Risk
OS: Overall Survival
PBMC: Peripheral Blood Mononuclear Cell
PET: Positron Emission Tomography
PSA: Prostate Specific Antigen
PSMA: Prostate Specific Membrane Antigen
QoL: Quality of Life
RFS: Recurrence-Free Survival
RLT: Radioligand Therapy
RP: Radical Prostatectomy
SBRT: Stereotactic Body Radiation Therapy
SPECT: Single Photon Emission Computed Tomography
SUV: Standard Uptake Value
TCR-seq: T-Cell Receptor Deep Sequencing
TRUS: Transrectal Ultrasound
utDNA: Urine Tumor Deoxyribonucleic Acid
ULN: Upper Limit of Normal
